# The role of multiple metabolic genes in predicting the overall survival of colorectal cancer: A study based on TCGA and GEO databases

**DOI:** 10.1371/journal.pone.0251323

**Published:** 2021-08-16

**Authors:** Weijun Shi, Xincan Li, Xu Su, Hexin Wen, Tianwen Chen, Huazhang Wu, Mulin Liu

**Affiliations:** 1 Department of Gastroenterology, First Affiliated Hospital of Bengbu Medical College, Bengbu, China; 2 Department of General Medicine, Second Affiliated Hospital of Bengbu Medical College, Bengbu, China; 3 School of Life Sciences, Anhui Province Key Laboratory of Translational Cancer Research, Bengbu Medical College, Bengbu, China; University of Nebraska Medical Center, UNITED STATES

## Abstract

The recent advances in gene chip technology have led to the identification of multiple metabolism-related genes that are closely associated with colorectal cancer (CRC). Nevertheless, none of these genes could accurately diagnose or predict CRC. The prognosis of CRC has been made by previous prognostic models constructed by using multiple genes, however, the predictive function of multi-gene prognostic models using metabolic genes for the CRC prognosis remains unexplored. In this study, we used the TCGA-CRC cohort as the test dataset and the GSE39582 cohort as the experimental dataset. Firstly, we constructed a prognostic model using metabolic genes from the TCGA-CRC cohort, which were also associated with CRC prognosis. We analyzed the advantages of the prognostic model in the prognosis of CRC and its regulatory mechanism of the genes associated with the model. Secondly, the outcome of the TCGA-CRC cohort analysis was validated using the GSE39582 cohort. We found that the prognostic model can be employed as an independent prognostic risk factor for estimating the CRC survival rate. Besides, compared with traditional clinical pathology, it can precisely predict CRC prognosis as well. The high-risk group of the prognostic model showed a substantially lower survival rate as compared to the low-risk group. In addition, gene enrichment analysis of metabolic genes showed that genes in the prognostic model are enriched in metabolism and cancer-related pathways, which may explain its underlying mechanism. Our study identified a novel metabolic profile containing 11 genes for prognostic prediction of CRC. The prognostic model may unravel the imbalanced metabolic microenvironment, and it might promote the development of biomarkers for predicting treatment response and streamlining metabolic therapy in CRC.

## Introduction

CRC is the most common malignancy of the digestive system. According to the recent global cancer statistics, the rank of CRC in the list of malignancies has ascended from the third to the second position, and its high morbidity and mortality rate pose a significant challenge to global public health [1]. Currently, the treatment of CRC primarily relies on surgical resection, chemotherapy, and physical therapy [2]. However, early distant metastasis is a common manifestation of this malignancy. Several patients surpass the advanced cancer stage by the time they are diagnosed with CRC, and so they are not amenable to curative treatments such as surgical resection. At present, many adjuvant chemotherapy regimens have been developed for the treatment of patients with advanced CRC, however, due to the high recurrence rate of CRC, there is no precise and effective adjuvant therapy to treat CRC, which will adversely affect the prognosis of patients receiving treatment [3]. Previous studies have extensively investigated the diagnosis and treatment of CRC, but prognosis monitoring of CRC remains inadequately addressed. Furthermore, due to the high mutation rate in CRC, conventional diagnostic approaches have been ineffective in predicting the treatment effect and recurrence of CRC accurately. Therefore, exploring a reliable method to monitor the CRC prognosis has become a crucial part of the treatment strategy.

In the past, there have been many studies on the predictive analysis of the prognosis of CRC [4], and most research focuses on the traditional clinicopathological indicators (such as tumor size, tumor count, lymph node metastasis, vascular invasion, etc.) or single tumor-specific biomarkers (such as carcinoembryonic antigen CEA, carbohydrate antigen-CA199, etc.) [5–7]. To a certain extent, this can provide reference materials for evaluating the prognosis of tumors, however, due to the subjectivity in the collection of pathological data, the complexity and variability of tumor occurrence and development, only reference to clinicopathological data or a single tumor-specific marker cannot make a reliable prediction of tumor prognosis. Recently, more and more studies have found that the prognostic model established by combining multiple genes has shown more encouraging results in the prognosis prediction of CRC than traditional prediction standards [8, 9]. However, the majority of these genes are associated only with the onset of malignancy. Thus, they are beneficial in the early diagnosis of malignancies. Conversely, these genes do not demonstrate satisfactory performance in prediction of prognosis of the tumor progression since the tumor progression is associated with a high frequency of oncogenic mutations [10]. Therefore, the differentially regulated genes associated with the CRC progression for the effective prognosis of CRC need further investigation.

A recent study reported that the differential expression of multiple metabolic genes in CRC, but their functional role and mechanism involved in CRC could not be explained [11]. In the early 1920s, Otto Warburg observed that tumor cells derive the energy from the process of glycolysis to promote tumor cell proliferation [12]. Recent studies have shown that non-glucose metabolites such as glutamic acid, fatty acids, and so on undergo significant metabolic alterations in tumor cells [13, 14]. A few potential candidate genes involved in metabolic disorders have been investigated through the preclinical or clinical protocols which suggests that metabolic genes are constantly involved in CRC progression [15]. Therefore, coalescing multiple metabolic genes can provide a reliable indicator to monitor the prognosis of CRC. Besides, it can also identify a novel molecular target for the metabolic treatment of CRC.

## Materials and methods

### Data acquisition

In this research, it is necessary to analyze the gene expression data and clinicopathological data of CRC. Related research shows that the Cancer Genome Atlas (TCGA) and the Gene Expression Omnibus (GEO) databases have a large amount of complete data for research and analysis. Perl and R are two computer programming languages with rich functions. In this study, Perl software can be used to extract and merge data. The "json" package of perl software was used to extract gene expression data in TCGA. The "XML::Simple" package is used to analyze clinical data files and extract clinical pathological data such as patient gender, age, classification, and staging. The "warnings" package is used to intersect TCGA gene expression data and metabolic gene data to obtain metabolic gene expression data in TCGA; combine gene expression data and the survival data of the corresponding patients to; GSEA enrichment analysis expression Preparation of data sets and phenotypic data sets. R software can be used to complete statistical analysis and visualization of data. The "Limma" and "pheatmap" packages of R software are used for the analysis of differential expression of genes. The "survival" package is used for COX analysis, survival analysis, univariate and multivariate independent prognostic analysis. The "glmnet" and "survival" program packages are used for Lasso regression analysis. The "pheatmap" package is used to draw the risk curve. The "survivalROC" package is used to draw ROC curves. The "rms" program package is used for drawing nomograms. The "ggplot2" program package is used for the visualization of GSEA results.

The gene expression and clinical data were fetched from the Cancer Genome Atlas-Colorectal Cancer data set (TCGA-CRC), which contained 44 normal colorectal and 568 CRC samples. The GSE39582 dataset was taken from the Gene Expression Omnibus database (GEO), which encompassed a total of 19 normal colorectal and 566 CRC samples. Through further processing of deleting missing items in the clinicopathological data, a total of 392 cases of TCGA-CRC and 550 cases of GSE39582 were obtained (Table 1). The metabolism-related genes are obtained from the GSEA website: First, download all KEGG pathway related genes (KEGG gene sets, gene symbols option) from the GSEA website (https://www.gsea-msigdb.org/gsea/downloads.jsp), which contains a total of 186 KEGG pathways (41 pathways are metabolically related, and 145 pathways are non-metabolically related); then extract the metabolic pathways All genes in are metabolic-related genes (S1 Table), which are used as metabolic gene expression data sets for subsequent analysis [16–18].

**Table 1:** Clinicopathological data of CRC in TCGA-CRC and GSE39582 dataset.

Term	TCGA-CRC		GSE39582	
	Number of cases	Proportion	Number of cases	Proportion
age				
<50	46	0.117	66	0.12
≥50	346	0.883	484	0.88
gender				
Male	206	0.526	299	0.544
Female	186	0.474	251	0.456
stage				
I	67	0.171	40	0.073
II	160	0.408	257	0.467
III	103	0.263	194	0.353
IV	62	0.158	59	0.107
T				
T1	8	0.02	16	0.029
T2	66	0.168	47	0.085
T3	272	0.694	372	0.676
T4	46	0.117	115	0.209
N				
N0	235	0.599	309	0.562
N1	89	0.227	136	0.247
N2	68	0.173	105	0.191
M				
M0	330	0.842	490	0.891
M1	62	0.158	60	0.109
futime				
~1	138	0.352	50	0.091
1~3	178	0.454	131	0.238
3~5	44	0.112	123	0.224
5~	32	0.082	246	0.447
fustat				
dead	73	0.186	176	0.32
alive	319	0.814	374	0.68

### Determining the overlapping differentially expressed metabolic genes in TCGA-CRC and GSE39582

First, Perl is used to search for metabolic genes in the TCGA-CRC cohort to obtain genes related to CRC metabolism. Then, R software was used to take the intersection of CRC metabolic genes and GSE39582 cohort genes, that is, obtain the metabolic genes that exist simultaneously in TCGA-CRC and GEO39582 cohorts. A total of 776 metabolic genes were obtained through intersection analysis, of which 407 genes were down-regulated (LogFC <0), and 369 genes were over-expressed (LogFC> 0). Finally, the "Limma" program package of R software was applied to the analysis of differentially expressed metabolic genes in CRC, and the differential genes were screened with the filter conditions of Log | Fc |> 0.5, FDR <0.01 (In order to allow more genes to be considered in the construction process of the model, we choose a slightly lower tolerance of 0.5 instead of logFC 1 or 2 for the more stringent and commonly used critical value).

### Construction of the prognostic model

First, use the "caret" package of the R program to randomly divide the TCGA cohort into a training cohort (TCGA-train) and a test cohort (TCGA-test) (in order to obtain a more reliable model, the data set is grouped at a ratio of 6:4). Univariate Cox regression analysis was applied to the differential gene expression and patient survival data of the TCGA-train cohort to screen metabolic genes related to patient prognosis. p<0.05 was used as a filtering criterion. Then, Lasso regression analysis is used to remove the more relevant genes to prevent the model from overfitting. Finally, multivariate Cox regression analysis is used to screen out genes that can be used as independent risk factors for the prognosis of CRC and calculate their corresponding risk coefficients, and build a metabolic model based on the risk coefficients [19]. The prognostic gene signature was shown as risk score = (Coefficient_mRNA1_ × expression of mRNA1) + (Coefficient_mRNA2_ × expression of mRNA2) + ⋯ + (Coefficient_mRNAn_ × expression mRNAn). Then, analyze the training set and build a metabolic model; and further cross-validate the model with the TCGA-test cohort.

### Validation of the accuracy of the prognostic model for predicting the prognosis of patients

The "survival ROC" package of R software was used to study the time dependence of the prognostic gene signature containing metabolic genes after evaluating the prognosis of CRC. The two-way log-rank test p<0.05 was of great significance for survival analysis. The "pheatmap" package was used to plot the risk curve to assess the predictive value of the risk score of the CRC prognosis metabolic gene prognostic model. The risk heat map was constructed to demonstrate the differential expression of each metabolic gene in the prognostic model between the high-risk group and the low-risk group. The risk curve and survival status chart were plotted to depict the correlation between the risk value of each sample with the risk groups and the patient’s survival status, respectively.

### Evaluation of the accuracy of the prognostic model

The accuracy of the prognostic model is verified by Concordance index (C-index), Receiver Operating Characteristic curve (ROC curve) and calibration curve. C-index is a model evaluation index, which is mainly used to calculate the difference between the predicted value of the COX model and the true value in the survival analysis [20]. When the C-index is equal to 0.5, the model has no predictive ability; when the C-index is 0.51–0.70, the accuracy is low; when the C-index is 0.71–0.90, the accuracy is medium; when it is greater than 0.90, the accuracy is high. The ROC curve is an exhaustive indicator that reflects continuous variables such as sensitivity and specificity. It can reveal the relationship between sensitivity and specificity (horizontal axis: false positive probability, vertical axis: true positive probability). The area under the curve was positively correlated to the accuracy. The "survival ROC" package was employed to perform ROC analysis of the prognostic model risk value and clinicopathological characteristics to assess the accuracy and sensitivity of the prognostic model for assessing the prognosis of CRC patients [21]. When AUC is equal to 0.5, the model has no predictive ability; 0.51–0.70, low accuracy; 0.71–0.90, medium accuracy; greater than 0.90, high accuracy. The calibration chart is to evaluate the accuracy of the model based on the degree of deviation between the calibration curve and the standard curve (the more the calibration curve deviates from the standard curve, the worse the prediction effect, and the closer it is to the standard curve, the better the prediction effect) [22]. The C-index and calibration curve are all analyzed and visualized by using the "rms", "foreign" and "survival" packages of the R software, while the ROC curve "survival ROC" package is used for analysis and visualization.

### Survival analysis of the prognostic model

The R package "survival" and "survminer" were utilized to determine the optimal cut-off risk score and to plot the survival curve. The "surv_cutpoint" function uses the "survminer" R software package to calculate the optimal cut-off value to categorize the CRC subjects into high and low-risk groups. The two-sided log-rank p<0.05 was used to determine statistical significance for survival analysis.

### Analysis of the prognostic effects of the prognostic model and clinicopathological features

The "survival" package was used for univariate and multivariate Cox regression analysis of the clinicopathological data and prognostic model risk scores of the TCGA-CRC and GSE39582 cohort. P<0.05 was used to determine statistical significance.

### Nomogram of the prognostic model

On the basis of the multivariate COX regression model, each gene in the model is listed on a flat diagram by using a scaled line segment according to a certain scale. Then, each gene is scored and the scores of all genes are added to obtain a total score, which is used to predict the survival and prognosis of CRC patients [23]. The nomogram is drawn using the "rms" package of R software.

### Gene set enrichment analysis

The TCGA-CRC and GSE39582 cohort were analyzed using GSEA to unravel the functional mechanism of genes included in the prognostic model. Perl software was used to prepare expression dataset files and phenotypic data files for gene enrichment analysis, for which the GSEA (http://software.broadinstitute.org/gsea) software and Java 8 runtime environment was employed. The results were visualized using R (plyr, ggplot2, grid, GridExtra package) software. P<0.05 and FDR<0.05 were used to determine statistical significance.

## Results

### Construction of the prognostic model in TCGA

**Fig 1:**
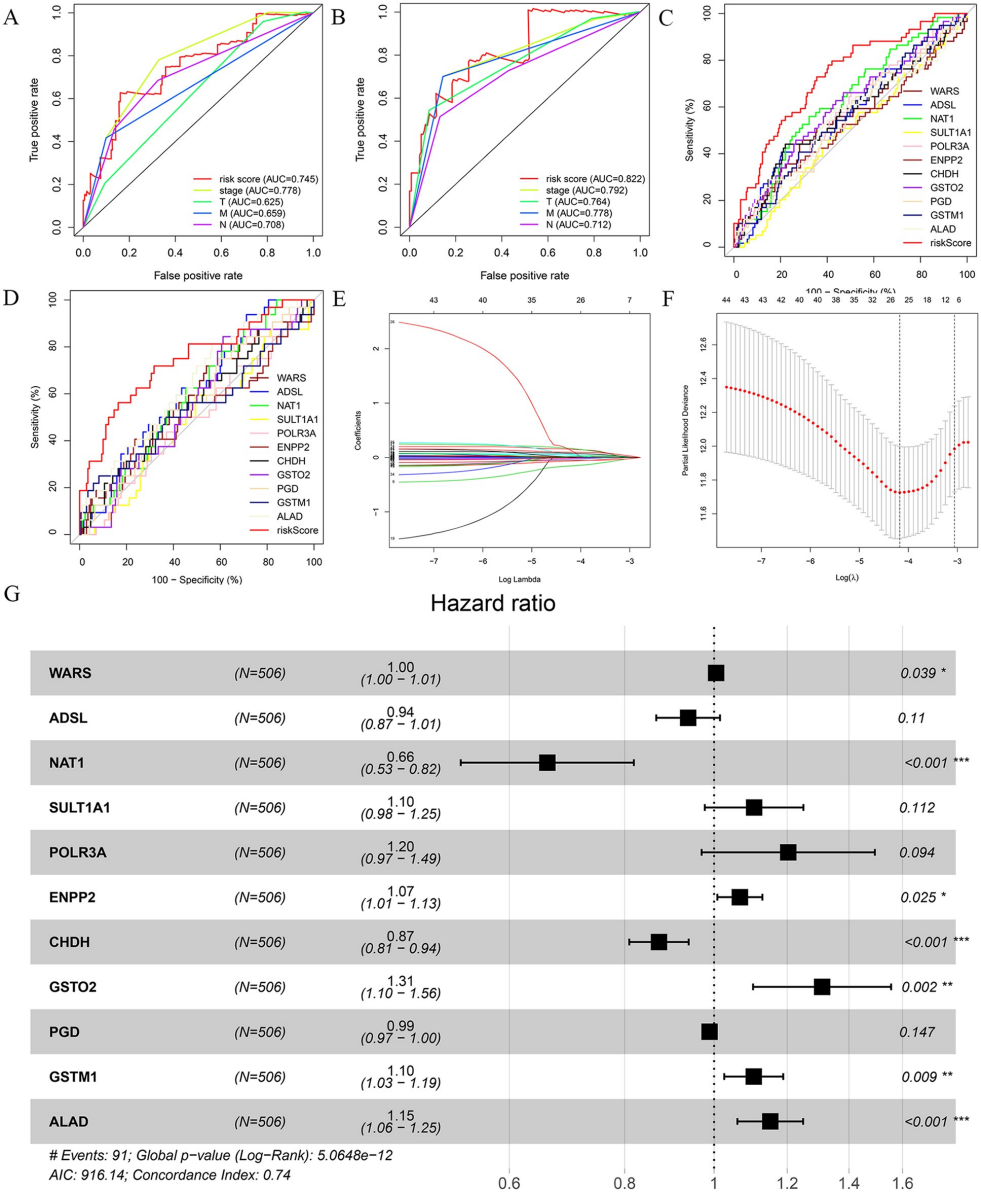
Screening and ROC analysis of the genes used for model construction. The ROC analysis of training and test data from TCGA-train (A and C) and TCGA-test cohort (B and D) showed that the combined multi-gene prognostic model is more accurate than clinicopathological (A and B) and single-gene (C and D). Lasso regression analysis (E and F), 25 genes were selected. “Log Lambda” represents the minimal cross-validation error. Multivariate Cox analysis (G) screened 11 genes for the construction of the prognostic model.

First of all, in the TCGA-train cohort, 44 genes related to the prognosis of colorectal cancer were screened out by univariate Cox regression analysis. Then, the Lasso regression analysis found that when the cross-validation error was the smallest (lambda = -4.2), 19 genes were eliminated due to strong correlation, and 25 genes were selected for subsequent analysis (Fig 1E and 1F). Finally, through multivariate Cox regression analysis, there are 11 genes (including 7 high-risk and 4 low-risk genes) that can be used as independent prognostic risk factors for colorectal cancer (Fig 1G). A prognostic model was constructed according to the risk coefficient of each gene (Table 2), and the prognostic model was displayed through the risk score. The risk score was calculated using the following formula: 0.005 × expression of WARS—0.065 × expression of ADSL-0.416 × expression of NAT1 + … + 0.14 × expression of ALAD. The ROC analysis of TCGA-train and TCGA-test cohort revealed that the prognostic model constructed by combining 11 genes had better accuracy in predicting the prognosis of colorectal cancer than clinicopathology and single genes (Fig 1A–1D, Table 3).

**Table 2:** The genes used to construct the model and their corresponding risk coefficient.

ID	coef	HR	HR.95L	HR.95H	P-value
WARS	0.005	1.005	1.000	1.010	0.039
ADSL	-0.065	0.937	0.866	1.015	0.110
NAT1	-0.416	0.660	0.532	0.819	0.000
SULT1A1	0.1	1.105	0.977	1.250	0.112
POLR3A	0.185	1.203	0.969	1.493	0.094
ENPP2	0.064	1.066	1.008	1.128	0.025
CHDH	-0.137	0.872	0.809	0.939	0.000
GSTO2	0.269	1.309	1.102	1.555	0.002
PGD	-0.011	0.989	0.974	1.004	0.147
GSTM1	0.099	1.104	1.026	1.188	0.009
ALAD	0.14	1.150	1.060	1.249	0.001

Note: "Coef" represents the risk coefficient of the corresponding gene. Positive values represent high-risk genes, and negative values represent low-risk genes.

**Table 3:** ROC analysis of train and test cohort.

Gene	TCGA-train			TCGA-test		
	AUC	Sensitivity	Specificity	AUC	Sensitivity	Specificity
WARS	0.545	0.458	0.706	0.573	0.719	0.459
ADSL	0.571	0.407	0.796	0.616	0.938	0.288
NAT1	0.626	0.559	0.678	0.592	0.781	0.394
SULT1A1	0.493	0.492	0.563	0.490	0.594	0.506
POLR3A	0.573	0.661	0.498	0.498	0.438	0.629
ENPP2	0.526	0.254	0.857	0.521	0.406	0.765
CHDH	0.570	0.441	0.780	0.563	0.469	0.688
GSTO2	0.601	0.458	0.735	0.554	0.844	0.388
PGD	0.549	0.644	0.478	0.567	0.531	0.635
GSTM1	0.564	0.407	0.722	0.539	0.250	0.918
ALAD	0.555	0.593	0.567	0.640	0.813	0.435
riskScore	0.732	0.797	0.580	0.740	0.719	0.682

### The prognostic model is accurate for the grouping of CRC patients

**Fig 2:**
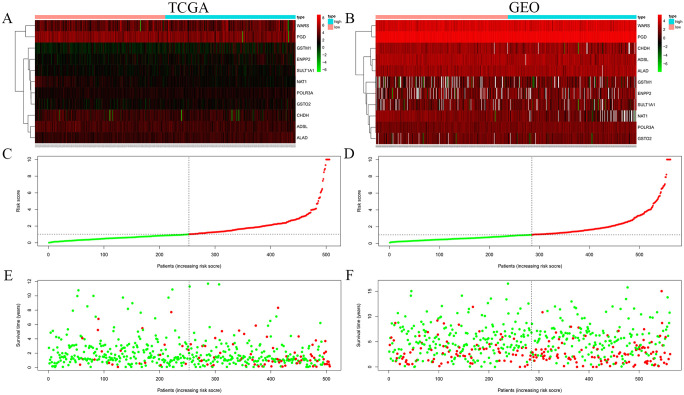
Risk heat map, risk curve and survival status diagram of risk score. The risk heat maps (A, B) show that 9 genes are highly expressed in the high-risk group and 4 genes are highly expressed in the low-risk group. The risk curves (C, D) show that the model risk score is positively correlated with the risk value of CRC patients. The survival status diagram (E, F) shows that people with higher risk values have a worse prognosis. A, C, and E are the analysis of the TCGA-CRC cohort, while B, D, and F are the analysis of the GSE39582 cohort.

In order to verify whether the prognostic model can accurately group CRC patients, we evaluated the correlation between the risk score of the prognostic model and the patient’s risk value and patient survival status. The risk plots demonstrated overexpression of 7 and 4 metabolic genes in the high-risk and low-risk group, respectively (Fig 2A and 2B). The risk score of this model can also be used to group patients accurately (Fig 2C and 2D). Besides, the survival status of high-risk patients was found to be poor as compared to low-risk patients (Fig 2E and 2F). Identical outcomes were shown by the TCGA-CRC (Fig 2A, 2C and 2E) and GSE39582 cohort (Fig 2B, 2D and 2F). Concisely, the risk score of the prognostic model has certain advantages in predicting the prognosis of patients.

### This prognostic model accurately predicts the prognosis of CRC patients

**Fig 3:**
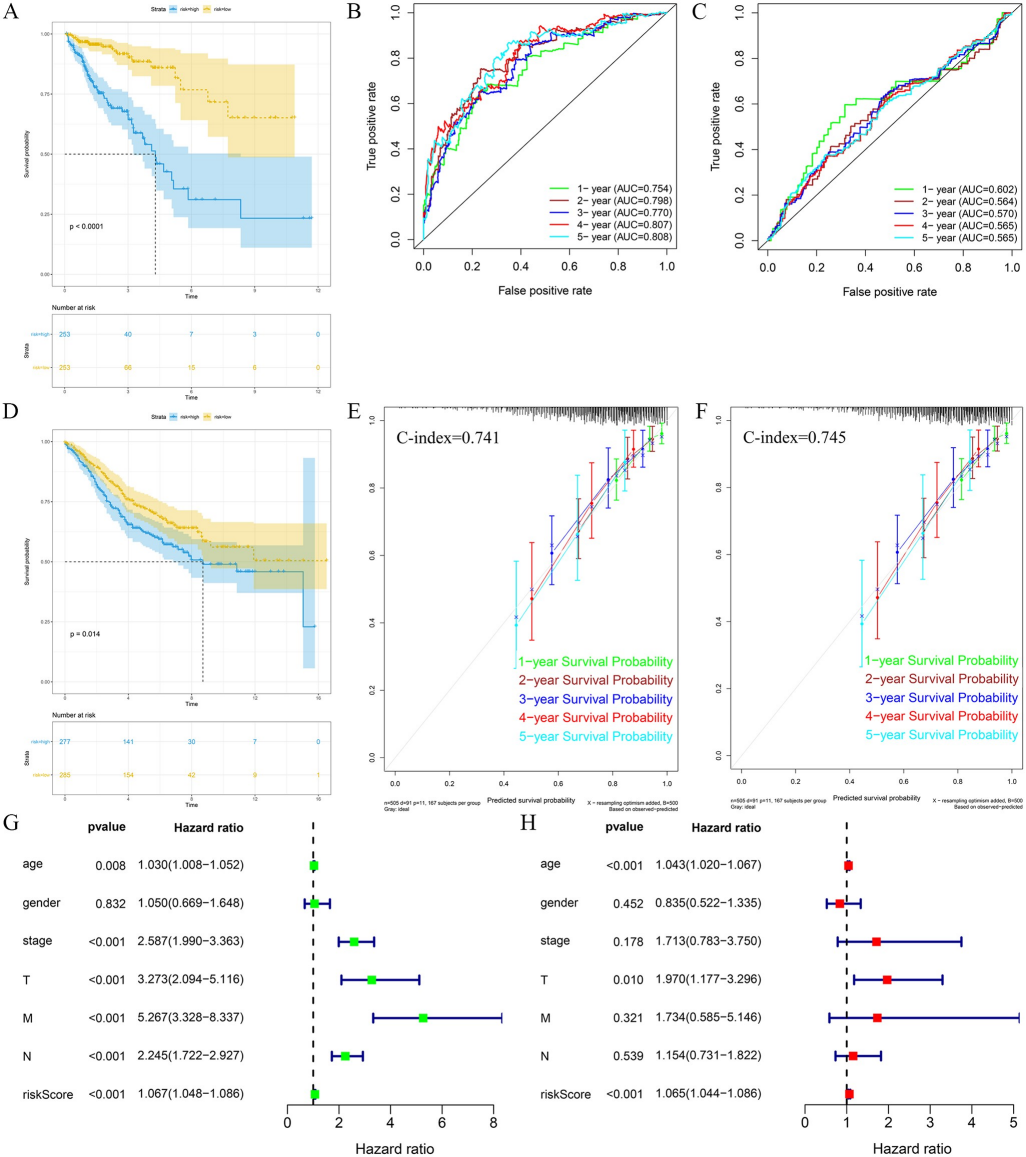
Survival analysis, independent prognostic analysis and verification of the model. The survival analysis of the TCGA-CRC(A) and GSE39582(D) cohorts showed that the overall survival rate of patients in the high-risk group was lower than that in the low-risk group. The ROC analysis of the TCGA-CRC(B) and GSE39582(C) cohorts showed that the prognostic model used to predict the prognosis of CRC is accurate. The C-index and calibration curve analysis of the TCGA-CRC(E) and GSE39582(F) cohorts showed that the prognostic model has strong accuracy in predicting the prognosis of CRC. Univariate (G) and multivariate (H) COX analysis of the TCGA-CRC cohort showed that the prognostic model can be used as an independent risk factor to predict the prognosis of CRC.

In order to verify the accuracy of the prognostic model for predicting the prognosis of CRC, we analyzed the prognostic model through the C-index, ROC curve and calibration curve. The prognostic model was found to have medium accuracy in both TCGA-CRC and GSE39582 queues through C-index validation, with their C-indexes of 0.741 and 0.745, respectively. The ROC curve analysis revealed that the prognostic model had medium accuracy (AUC > 0.75) for the 1-, 2-, 3-, 4-, and 5-year prognosis in the TCGA-CRC cohort and relatively poor accuracy in the GSE39582 cohort (Fig 3B and 3C). Calibration plot analysis showed that the calibration curves and standard curves of the TCGA-CRC and GSE39582 cohorts exhibited high aggregation, which indicated that the prognostic model had high accuracy in predicting the CRC prognosis (Fig 3E and 3F). In summary, this prognostic model can be used accurately for the prediction of survival and prognosis of CRC patients.

### High risk score indicates poor prognosis for CRC

To analyze the role of prognostic models in predicting CRC prognosis, we performed a survival analysis of the patients’ risk scores. Through the risk score of the prognostic model, patients were divided into a high-risk group (risk score > median risk score) and a low-risk group (risk score < median risk score). The results showed that the overall survival time in the high-risk group was substantially lower than that in the low-risk group (P<0.05), and both the TCGA-CRC and GSE39582 cohorts exhibited the same results (Fig 3A and 3D).

### The prognostic model can be used as an independent risk signal for the prognosis of CRC

In order to further analyze if the prognostic model can be used as an independent risk factor for evaluating the prognosis of CRC, we conducted univariate and multivariate COX analysis of the prognostic model and clinicopathological characteristics [24]. Univariate (Fig 3G) and multivariate (Fig 3H) Cox regression analysis of 392 subjects in the TCGA-CRC cohort showed that age, TNM stage and prognostic metabolic gene markers developed in this study can be used to assess overall survival (OS) Independent prognostic factors, but the results of the analysis of the GSE39582 cohort are relatively poor. The analysis of independent prognosis revealed that the prognostic model can be used as an independent risk factor for the evaluation of the prognosis of CRC.

### Nomogram of prognostic model

**Fig 4:**
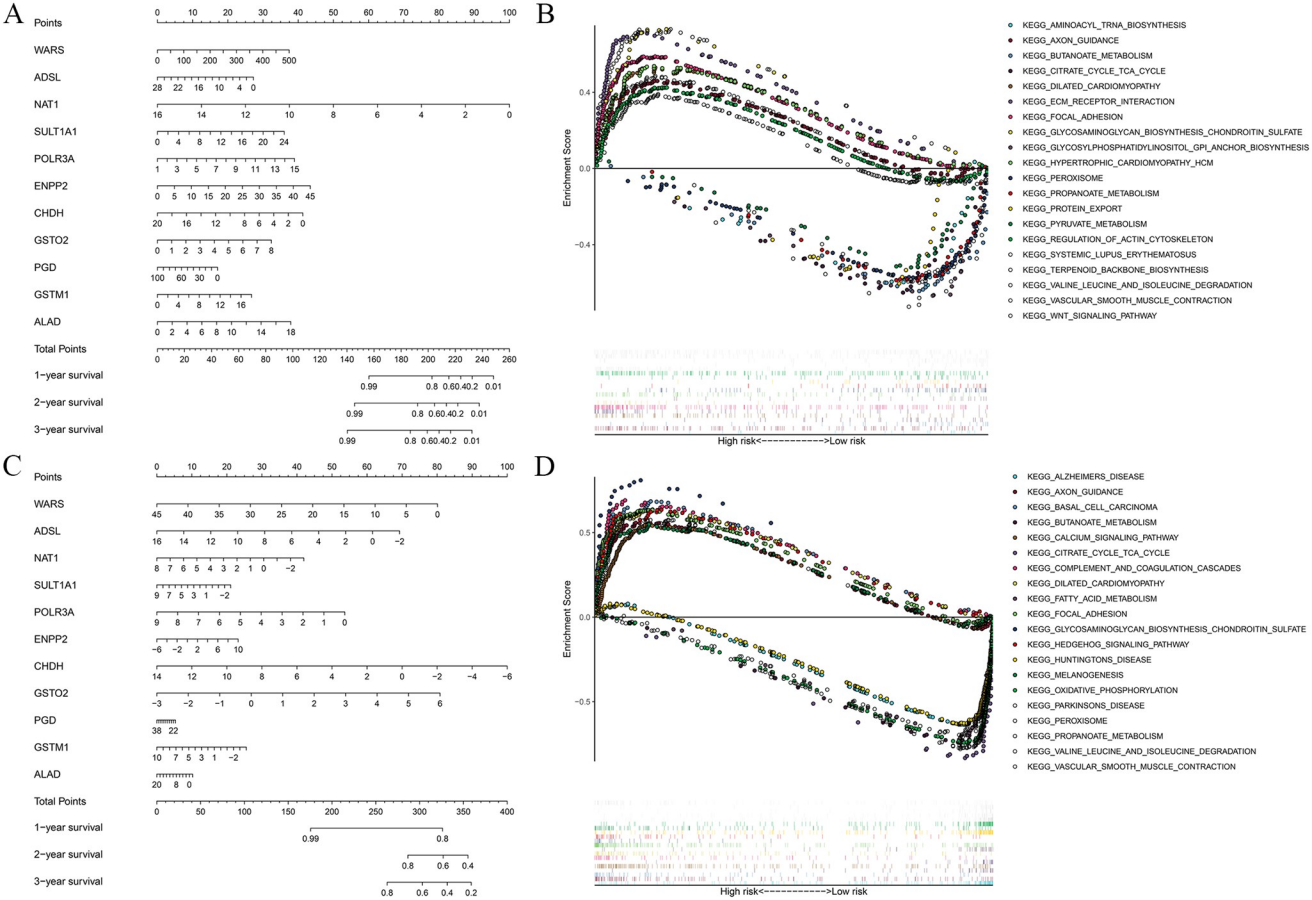
Nomogram and GSEA analysis. The nomograms of the TCGA-CRC(A) and GSE39582(C) cohorts show that each gene in the prognostic model has a different contribution to the prediction of prognosis. In addition, compared with the long-term survival period (2 or 3 years), the prognostic model has better predictive value for the short-term survival period (1 year). GESA analysis of the TCGA-CRC(B) and GSE39582(D) cohorts showed that the genes of the prognostic model were significantly enriched in metabolism and tumor-related signaling pathways.

In order to predict the occurrence probability of patients with different survival times, we draw a nomogram based on the relationship between each gene in the prognostic model and the different survival rates of colorectal cancer patients. The nomogram shows that the gene NAT1 in the TCGA-CRC cohort (Fig 4A) and the gene CHDH in the GSE39582 cohort (Fig 4C) play a key role in the prediction of the model. Gene PGD in the TCGA-CRC and GSE39582 cohort, its contribution to the prognostic model is small. Further analysis found that the prognostic model has better predictive value for patients’ short-term survival (1 year) than long-term survival (2 or 3 years).

### Gene set enrichment analysis of metabolic genes

In the GSEA of metabolic genes, TCGA-CRC and GSE39582 cohort showed significant enrichment of 86 and 22 KEGG pathways, respectively. A majority of these pathways were either related to metabolic processes such as the metabolism of glucose, fatty acids, proteins, and nucleic acids or metabolic disorders such as diabetes, Parkinson’s disease, and so on. The rest of the pathways were not related to metabolism, but were often deregulated either in different cancer types such as basal cell carcinoma, small cell lung cancer, and acute myeloid leukemia or in cancer-related signaling pathways such as NOTCH, MAPK, JAK_STAT, and WNT pathways. Furthermore, the majority of these metabolism-related pathways were significantly enriched in the low-risk group, while most of the pathways not related to metabolism were enriched in the high-risk group (Fig 4B and 4D, Tables 4 and 5).

**Table 4:** GSEA enrichment analysis results in the TCGA-CRC cohort (top 20).

NAME	ES	NES	P-value
KEGG_BASAL_CELL_CARCINOMA	0.685	2.377	0.000
KEGG_COMPLEMENT_AND_COAGULATION_CASCADES	0.691	2.278	0.000
KEGG_DILATED_CARDIOMYOPATHY	0.637	2.205	0.000
KEGG_HEDGEHOG_SIGNALING_PATHWAY	0.635	2.199	0.000
KEGG_GLYCOSAMINOGLYCAN_BIOSYNTHESIS_CHONDROITIN_SULFATE	0.810	2.178	0.000
KEGG_VASCULAR_SMOOTH_MUSCLE_CONTRACTION	0.597	2.229	0.000
KEGG_FOCAL_ADHESION	0.634	2.217	0.002
KEGG_CALCIUM_SIGNALING_PATHWAY	0.546	2.179	0.000
KEGG_AXON_GUIDANCE	0.558	2.140	0.002
KEGG_MELANOGENESIS	0.549	2.130	0.000
KEGG_PARKINSONS_DISEASE	-0.779	-2.398	0.000
KEGG_PEROXISOME	-0.704	-2.372	0.000
KEGG_ALZHEIMERS_DISEASE	-0.640	-2.353	0.000
KEGG_HUNTINGTONS_DISEASE	-0.636	-2.349	0.000
KEGG_FATTY_ACID_METABOLISM	-0.738	-2.341	0.000
KEGG_PROPANOATE_METABOLISM	-0.776	-2.330	0.000
KEGG_VALINE_LEUCINE_AND_ISOLEUCINE_DEGRADATION	-0.792	-2.285	0.000
KEGG_CITRATE_CYCLE_TCA_CYCLE	-0.850	-2.262	0.000
KEGG_OXIDATIVE_PHOSPHORYLATION	-0.749	-2.247	0.000
KEGG_BUTANOATE_METABOLISM	-0.764	-2.253	0.000

**Table 5:** GSEA enrichment analysis results in the GSE39582 cohort (top 20).

NAME	ES	NES	P-value
KEGG_ECM_RECEPTOR_INTERACTION	0.715	1.680	0.010
KEGG_AXON_GUIDANCE	0.460	1.589	0.014
KEGG_FOCAL_ADHESION	0.589	1.723	0.016
KEGG_GLYCOSAMINOGLYCAN_BIOSYNTHESIS_CHONDROITIN_SULFATE	0.731	1.659	0.016
KEGG_HYPERTROPHIC_CARDIOMYOPATHY_HCM	0.538	1.619	0.025
KEGG_DILATED_CARDIOMYOPATHY	0.530	1.603	0.029
KEGG_REGULATION_OF_ACTIN_CYTOSKELETON	0.425	1.566	0.034
KEGG_VASCULAR_SMOOTH_MUSCLE_CONTRACTION	0.485	1.534	0.036
KEGG_SYSTEMIC_LUPUS_ERYTHEMATOSUS	0.731	1.561	0.038
KEGG_WNT_SIGNALING_PATHWAY	0.391	1.402	0.039
KEGG_GLYCOSYLPHOSPHATIDYLINOSITOL_GPI_ANCHOR_BIOSYNTHESIS	-0.708	-1.879	0.000
KEGG_CITRATE_CYCLE_TCA_CYCLE	-0.674	-1.915	0.002
KEGG_PEROXISOME	-0.587	-1.767	0.008
KEGG_TERPENOID_BACKBONE_BIOSYNTHESIS	-0.755	-1.739	0.010
KEGG_PROTEIN_EXPORT	-0.643	-1.746	0.010
KEGG_BUTANOATE_METABOLISM	-0.659	-1.616	0.012
KEGG_PROPANOATE_METABOLISM	-0.610	-1.667	0.017
KEGG_PYRUVATE_METABOLISM	-0.537	-1.632	0.019
KEGG_VALINE_LEUCINE_AND_ISOLEUCINE_DEGRADATION	-0.611	-1.635	0.029
KEGG_AMINOACYL_TRNA_BIOSYNTHESIS	-0.658	-1.583	0.039

## Discussion

Although the incidence and mortality rates of CRC have waned, as per the latest World Cancer Statistics (Cancer Statistics, 2020), CRC has ascended to the third position in the global malignancy list [25]. As compared to the studies on the diagnosis and treatment of CRC, only a handful of studies have been conducted in CRC clinical prognosis. Earlier, the clinical prediction of prognosis was primarily based on clinicopathological characteristics such as age, pathological grade, and TNM staging. As these clinicopathological characteristics are highly biased, it is difficult to make a precise prediction of the prognosis based on them [26, 27]. In recent years, research studies based on the mRNA characteristic (such as alpha-fetoprotein in liver cancer and prostate-specific antigen in prostate cancer, and so on) for predicting the mortality risk of cancer has become a new hotspot for researchers, but so far, none of these studies were able to identify reliable indicators for the prediction of CRC prognosis [28].

In the current study, for the prediction of CRC prognosis, the prognostic model containing 11 metabolic genes were recognized, and its effectiveness was verified in the GSE39582 cohort. Analysis of risk curves, C-index, ROC curves and calibration plots of the prognostic model showed that the prognostic model could be effectively stratified for patient survival and accurately used to predict the prognosis of CRC patients. Independent prognostic and survival analyses showed that this prognostic model could be used as an independent risk factor to evaluate patient prognosis and that the high-risk group had a worse prognosis than the low-risk group. In addition, we found that the predictive value analysis of the GSE39582 cohort was poor as compared to the TCGA-CRC cohort. It may be due to the variation in the baseline characteristics of the subjects in the TCGA-CRC and GSE39582 cohort or due to the variation in the sample of the GSE39582 cohort as they belonged to different batches. The results validated the higher prognostic value of our prognostic model; however, it demands further validation in a more independent database.

Furthermore, GSEA analysis revealed that metabolic genes were significantly enriched in different pathways, most of which were related to metabolism and cancer. These outcomes further validated the close relationship between the prognostic model and the metabolic system. Besides, it indicated that genes associated with metabolic processes could promote the onset and progression of cancer through the regulation of cancer-associated metabolic pathways. Interestingly, we found that low-risk scores were primarily correlated with metabolic pathways, while high-risk scores were primarily correlated with non-metabolic pathways such as immunity, cancer, and cancer-related pathways. Thus, metabolic therapy can be used for treating patients with low-risk scores, while immunotherapy can be used for treating patients with high-risk scores [29, 30]. These outcomes may unravel the underlying molecular mechanism of gene signatures associated with the prediction of CRC prognosis. However, a further in-depth investigation is required to establish the relationship between genes involved in this model and metabolic microenvironment as well as the metabolic therapy in CRC. In Conclusion, the prognostic gene signature identified in our study may represent metabolic microenvironment disorders, and it might lead to the development of biomarkers for predicting treatment response and defining metabolic therapy in CRC patients.

According to the previous studies, the 11 genes in the prognostic model are associated with the metabolic enzymes and play a crucial role in multiple metabolic pathways such as glucose metabolism (CHDH, PGD) [31], fatty acid metabolism (NAT1) [32], protein metabolism (WARS, SULT1A1, GSTO2 and GSTM1) and nucleotide metabolism (ADSL, POLR3A, ENPP2) [33]. In addition, we found that most of the genes are associated with cancer initiation, cell proliferation and metastasis. For example, Tryptophanyl-tRNA synthetase (WARS) is low expressed in colorectal cancer tissues and can promote tumor metastasis by promoting angiogenesis via the VEGF and p53 signaling pathways [34]. N-acetyltransferase 1 (NAT1) acts as a direct target of miR-6744-5p and promotes the Distant metastasis in triple-negative breast cancer [35]. Sulfotransferase 1A1 (SULT1A1) is a drug and hormone metabolizing enzyme involved in the metabolism of a variety of endogenous and exogenous potential breast carcinogens, the absence of which can lead to the development of male breast cancer [36]. In bladder cancer, RNA polymerase III (POLR3A) promotes the proliferation of bladder cancer cells by inhibiting their differentiation; in colorectal cancer, POLR3A is expressed at high levels and is associated with advanced tumor depth, lymph node metastasis, distant metastasis, and poor prognosis [37, 38]. Lead exposure leads to increased aberrations in the methylation of Aminolevulinate delta dehydratase (ALAD) and p16 genes and ultimately to lead poisoning [39].

The current study primarily focused on the determination of the prognostic role of metabolic genes in CRC, not limited to a single metabolic gene or pathway. Despite the detailed and rigorous analysis and substantial clinical significance, this study is subject to certain limitations. Firstly, in this study, only 776 metabolic genes were used to evaluate the prognostic model. Thus, we speculate that some crucial prognostic metabolic genes may have been excluded in the process, which might hamper the efficiency of this model. Secondly, the onset and progression of CRC is a complex process where multiple mechanisms work together. The comparatively poor performance of the metabolic gene prognosis model indicates that the CRC prognosis model established only by metabolic genes is insufficient to evaluate the prognosis of CRC. Lastly, the model needs experimental validation to unravel the complex mechanisms of metabolic genes included in the prognostic model.

## Conclusion

In the current study, we constructed a novel prognostic model containing 11 genes for CRC prognosis prediction based on the TCGA-CRC cohort, and verified its high prognostic value using the GSE39582 cohort. This prognostic model reflected the dysregulation of the metabolic microenvironment and the regulatory pathways in CRC. Besides, it unraveled that the risk score of the prognostic model can be used not only to predict the therapeutic effect of CRC but also to provide a basis for the selection of metabolic therapy or immunotherapy. However, this prognostic model demands further validation in a more independent database along with the experimental validation.

## Supporting information

S1 Table 41 metabolic pathways used to extract metabolism-related genes. (DOCX)

S1 Database (TXT)

S2 Database (TXT)

S3 Database (TXT)

S4 Database (TXT)
