# Supplementary material for: The role of multiple metabolic genes in predicting the overall survival of colorectal cancer: A study based on TCGA and GEO databases
Source: PLoS One. 2021 Aug 16;16(8):e0251323. doi: 10.1371/journal.pone.0251323 (PMC8367004; doi:10.1371/journal.pone.0251323)
Supplement: S1 Table — (DOCX) [file pone.0251323.s001.docx]

**S1 Table 41 metabolic pathways used to extract metabolism-related genes**

| KEGG_FRUCTOSE_AND_MANNOSE_METABOLISM |
| --- |
| KEGG_GALACTOSE_METABOLISM |
| KEGG_ASCORBATE_AND_ALDARATE_METABOLISM |
| KEGG_FATTY_ACID_METABOLISM |
| KEGG_PURINE_METABOLISM |
| KEGG_PYRIMIDINE_METABOLISM |
| KEGG_ALANINE_ASPARTATE_AND_GLUTAMATE_METABOLISM |
| KEGG_GLYCINE_SERINE_AND_THREONINE_METABOLISM |
| KEGG_CYSTEINE_AND_METHIONINE_METABOLISM |
| KEGG_ARGININE_AND_PROLINE_METABOLISM |
| KEGG_HISTIDINE_METABOLISM |
| KEGG_TYROSINE_METABOLISM |
| KEGG_PHENYLALANINE_METABOLISM |
| KEGG_TRYPTOPHAN_METABOLISM |
| KEGG_BETA_ALANINE_METABOLISM |
| KEGG_TAURINE_AND_HYPOTAURINE_METABOLISM |
| KEGG_SELENOAMINO_ACID_METABOLISM |
| KEGG_GLUTATHIONE_METABOLISM |
| KEGG_STARCH_AND_SUCROSE_METABOLISM |
| KEGG_AMINO_SUGAR_AND_NUCLEOTIDE_SUGAR_METABOLISM |
| KEGG_GLYCEROLIPID_METABOLISM |
| KEGG_INOSITOL_PHOSPHATE_METABOLISM |
| KEGG_GLYCEROPHOSPHOLIPID_METABOLISM |
| KEGG_ETHER_LIPID_METABOLISM |
| KEGG_ARACHIDONIC_ACID_METABOLISM |
| KEGG_LINOLEIC_ACID_METABOLISM |
| KEGG_ALPHA_LINOLENIC_ACID_METABOLISM |
| KEGG_SPHINGOLIPID_METABOLISM |
| KEGG_PYRUVATE_METABOLISM |
| KEGG_GLYOXYLATE_AND_DICARBOXYLATE_METABOLISM |
| KEGG_PROPANOATE_METABOLISM |
| KEGG_BUTANOATE_METABOLISM |
| KEGG_RIBOFLAVIN_METABOLISM |
| KEGG_NICOTINATE_AND_NICOTINAMIDE_METABOLISM |
| KEGG_RETINOL_METABOLISM |
| KEGG_PORPHYRIN_AND_CHLOROPHYLL_METABOLISM |
| KEGG_NITROGEN_METABOLISM |
| KEGG_SULFUR_METABOLISM |
| KEGG_METABOLISM_OF_XENOBIOTICS_BY_CYTOCHROME_P450 |
| KEGG_DRUG_METABOLISM_CYTOCHROME_P450 |
| KEGG_DRUG_METABOLISM_OTHER_ENZYMES |
